# Zinc Oxide Exerts Anti-Inflammatory Properties on Human Placental Cells

**DOI:** 10.3390/nu12061822

**Published:** 2020-06-18

**Authors:** Andrea Balduit, Alessandro Mangogna, Chiara Agostinis, Gabriella Zito, Federico Romano, Giuseppe Ricci, Roberta Bulla

**Affiliations:** 1 Department of Life Sciences, University of Trieste, 34127 Trieste, Italy; abalduit@units.it (A.B.); alessandro.mangogna@studenti.units.it (A.M.); rbulla@units.it (R.B.); 2 Institute for Maternal and Child Health, IRCCS Burlo Garofolo, 34134 Trieste, Italy; gabriella.zito@burlo.trieste.it (G.Z.); federico.romano@burlo.trieste.it (F.R.); giuseppe.ricci@burlo.trieste.it (G.R.); 3 Department of Medical, Surgical and Health Science, University of Trieste, 34129 Trieste, Italy

**Keywords:** inflammation, zinc oxide, endothelium, inflammatory cytokines, dietary supplement, pregnancy

## Abstract

Background: An aberrant and persistent inflammatory state at the fetal-maternal interface is considered as a key contributor in compromised pregnancies. Decidual endothelial cells (DECs) play a pivotal role in the control of the local decidual inflammation. The aim of the current study was to determine whether dietary supplement with zinc oxide (ZnO), due to its very low adverse effects, may be useful for modulating the inflammatory response in the first trimester of pregnancy. Methods: The anti-inflammatory properties of ZnO in pregnancy were evaluated by in vitro tests on endothelial cells isolated from normal deciduas and on a trophoblast cell line (HTR8/Svneo). The effects of this treatment were analyzed in terms of adhesion molecule expression and inflammatory cytokine secretion, by real time-quantitative PCR (RT-qPCR) and enzyme-linked immunosorbent assay (ELISA). Results: Our data showed that ZnO was able to reduce the inflammatory response of DECs, in terms of vascular cell adhesion molecule-1 (VCAM-1), interleukin (IL)-8, IL-6, tumor necrosis factor-α (TNF-α) and monocyte chemoattractant protein-1 (MCP-1) expression induced by TNF-α stimulation. This compound exerted no effect on intracellular adhesion molecule-1 (ICAM-1) exocytosis induced by TNF-α on stimulated trophoblast cells, but significantly reduced their IL-6 expression. Conclusion: According to these results, it can be suggested that the ZnO supplement, through its modulation of the pro-inflammatory response of DECs, can be used in pregnancy for the prevention of local decidual inflammation.

## 1. Introduction

Although the role of inflammation is crucial and necessary for successful embryo implantation [1], an aberrant and persistent inflammatory state at the fetal-maternal interface is considered a key contributor in compromised pregnancies. Embryo implantation failure, occult pregnancy loss, recurrent miscarriage, pre-eclampsia, fetal growth restriction, and preterm birth are frequent disorders of normal human reproduction related to the dysregulation of the maternal inflammatory response [2]. The excessive pro-inflammatory state that characterizes adverse pregnancy outcome may to some extent arise from an increased production of pro-inflammatory cytokines, such as interleukin (IL)-6, IL-8 and tumor necrosis factor-α (TNF-α) and/or the decreased production of anti-inflammatory cytokines such as IL-4 and IL-10 [3]. Despite the fact that the mechanisms engaged in the prevention of excessive maternal immune activation are still not completely understood, the prompt lowering of inflammation during pregnancy may be critical in the prevention of pregnancy disorders [4].

**Figure 1:**
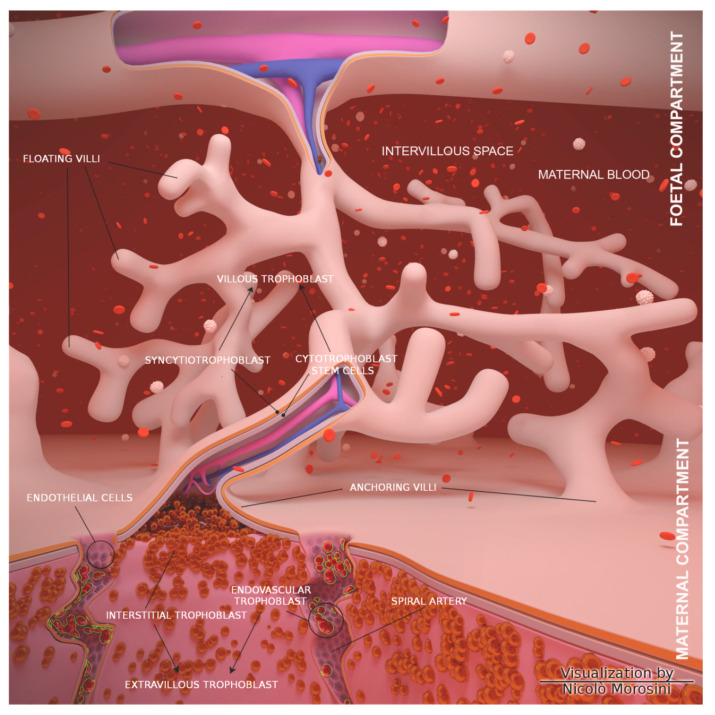
Schematic representation of the placenta morphology. Cartoon showing an overview of the structural organization of the placenta and fetal membranes (the image was designed using the graphic design free software Blender 3D Software, Blender Foundation, Stichting Blender Foundation, Buikslotermeerplein, Amsterdam, the Netherlands). The maternal-fetal interface is a direct contact between maternal (decidua) and fetal (chorion or trophoblast) tissues. Therefore, there is a separation between the maternal basal plate and the fetal plate, where the maternal spiral arteries wet the villous trees through the chorionic plate.

The human placenta is an invasive structure mainly composed of highly proliferative cytotrophoblast stem cells and invasive extravillous trophoblast (EVT), which migrates and invades the uterus wall (interstitial trophoblast) and its spiral arteries (endovascular trophoblast), partially replacing endothelial cells (ECs) (Figure 1). In humans, the placenta is hemochorial because maternal blood is in direct contact with the fetal trophoblast. In the feto-maternal interface, the presence of leukocytes is responsible for several functions, such as the limitation of trophoblast invasion, the remodeling of spiral arteries, the maintenance of maternal tolerance and the initiation of labor [5].

The ECs lining decidual vessels appear as pivotal players in the control of the inflammation at foeto-maternal interface [6]. ECs are capable of secreting a plethora of biologically active mediators and, during inflammation, they enhance the expression of cell adhesion molecules and cytokines, orchestrating the inflammatory response [6]. We focused our interest also on trophoblast cells which represent a large fraction of decidual cells, as well as the hallmark of the placenta. Besides covering the surface of the villi as villous trophoblasts, these cells migrate into the decidua as a distinct population of EVT and contribute to induce tissue remodeling and vascular changes in the decidua [7]. Furthermore, trophoblast cells are also able to increase the surface expression of intracellular adhesion molecule-1 (ICAM-1) in response to pro-inflammatory cytokines, such as TNF-α or IL-1β [8]. 

Woman’s nutritional status during pregnancy plays an important role in preventing pregnancy disorders, as a result of the potential benefits of nutritional supplements that regulate key targets related to oxidative stress and inflammation [9]. An adequate nutritional intake is essential to ensure appropriate growth and correct fetal development [10]. The Nordic Nutrition Recommendation (NNR) encourages pregnant women to improve their folate intake by 65%, selenium by 40%, iron, zinc and vitamin D by 25–30%, and most other minerals and vitamins by 20% [11]. During pregnancy, an increased intake of zinc is needed for its contribution to growth and development of unborn children, since an inadequate maternal zinc status is currently associated with poor pregnancy outcome, congenital malformations and preterm or post-term deliveries [12,13,14]. Furthermore, zinc contributes to the development of the fetal brain [15] and zinc deficiency has also been linked to autism spectrum disorders [16]. The metabolic need of zinc increases during gestation; in fact, for adults, 10 mg/day are normally required [17], whereas an intake of 15 mg/day has been suggested in pregnant women, assuming a bioavailability of 20–33% [18,19,20,21,22].

Based on these statements, the aim of the current study was to evaluate whether the dietary supplement with zinc oxide (ZnO) may modulate the inflammatory response of decidual endothelial cells (DECs) and trophoblast cells in the first trimester of pregnancy.

## 2. Materials and Methods

### 2.1. Reagents

ZnO (Sigma-Aldrich, Milan, Italy) was solubilized in 1 M HCl, in order to obtain an initial solution of 0.5 mg/mL, then filtered with a 0.22 µm syringe filter and stored at 4 °C until use. A fresh solution of ZnO was prepared for every experiment. The prepared solution was added to complete cell culture medium, in order to obtain a final concentration of 15 µg/mL and incubated at 37 °C in 5% *v/v* CO_2_ incubator. The effect of ZnO addition in terms of the change of cell culture medium pH was measured by pH-Meter Basic20+ (Crison Instrument Srl, Milan, Italy). The effect of ZnO addition in terms of cell viability was evaluated by adding the water soluble tetrazolium-1 (WST-1) reagent of the Quick Cell Proliferation Kit (Abcam, Cambridge, MA, USA) and reading the plate at 450 nm with a spectrophotometer.

### 2.2. Cell Isolation and Culture

ECs were isolated from the normal decidual tissues of women, following the method previously described [23]. Five women undergoing elective pregnancy termination (age ranging from 20 to 35 years) at the Institute for Maternal and Child Health, IRCCS Burlo Garofolo, Trieste, Italy, were enrolled. The study was reviewed and approved by the Regional Ethical Committee of FVG (CEUR), Udine, Italy (Prot. 2605/2019). Informed consent for participation in the study was obtained from all women. 

Briefly, the tissue was mechanically and enzymatically digested with 0.5% trypsin and then with 3 mg/mL collagenase I; the cells collected at the interface of Ficoll-Paque gradient (LifeTechnologies, Milan, Italy) were positively selected with Dynabeads M-450 (Dynal, Invitrogen, Milano, Italy) and coated with Ulex europaeus 1 lectin (UEA-1, Sigma-Aldrich). Cytofluorimetric analysis showed that more than 95% of the cells were positively stained for von Willebrand Factor (vWF;Dako, Milan, Italy). Cells were seeded in a pre-coated flask with 2 µg/cm^2^ fibronectin (Roche, Milan, Italy) and maintained in a endothelial serum-free basal medium (LifeTechnologies), supplemented with 20 ng/mL bFGF and 10 ng/mL EGF (LifeTechnologies). 

HTR8/SVneo, a human EVT cell line, kindly provided by Peeyush K. Lala (Department of Anatomy and Cell Biology, University of Western Ontario, Canada), were cultured in RPMI (LifeTechnologies), supplemented with 10% fetal calf serum (FCS). 

### 2.3. Immunofluorescence

DECs, labelled with the fluorescent dye FAST DiI (Molecular Probes, Invitrogen), were seeded onto 8-chamber culture slides (BD Biosciences Discovery Labware, Milan, Italy), previously coated with 2 µg/cm^2^ fibronectin (Roche). After fixation with 1% paraformaldehyde and permeabilization with FIX & PERM kit, solution B (Società Italiana Chimici, Rome, Italy), cells were incubated with 10 µg/mL primary monoclonal antibody (cloneF8/86) mouse anti-human vWF (Dako) or mouse anti-human vascular endothelial (VE)-cadherin (kindly provided by Prof. Dejana from the Institute of Molecular Oncology, Milan, Italy) for 1 h at room temperature (RT), followed by incubation with fluorescein isothiocyanate (FITC)-conjugated goat anti-mouse IgG (Dako) for 1h at RT. Images were acquired with a Leica DM3000 microscope (Leica, Wetzlar, Germany) and collected using a Leica DFC320 digital camera (Leica).

### 2.4. Enzyme-Linked Immunosorbent Assay (ELISA) for VCAM-1 or ICAM-1 on Whole Cells

Both ECs and HTR8/SVneo were grown up to confluence in 96-well plates (10^5^ cells/wells) and then treated with 15 µg/mL ZnO for 48 h at 37 °C in 5% *v/v* CO_2_ incubator. Subsequently, cells were stimulated overnight (ON) with 100 ng/mL TNF-α (Peprotech, Milan, Italy), and then incubated with 5 µg/mL mouse mAb anti-human vascular cell adhesion molecule-1 (VCAM-1, Sigma-Aldrich) for DECs or mAb anti-human ICAM-1 (Dako), for 90 min at RT. The binding of the primary antibody was quantified by incubating the cells with polyclonal anti-mouse IgG alkaline phosphatase (AP)-conjugated (Sigma-Aldrich; 1:10.000), for 1 h at RT. The enzymatic reaction was developed with 1 mg/mL para-nitrophenyl phosphate (PNPP, Sigma-Aldrich) as a substrate, and read kinetically at 405 nm using a Titertek Multiskan ELISA reader (Flow Labs, Milano, Italy). The resting condition was represented by DECs’ basal expression of VCAM-1 (or trophoblasts’ ICAM-1), without any stimulation or treatment, whilst the untreated condition was considered as cells stimulated with TNF-α, but not with ZnO. The untreated condition was considered as our reference value of 100%. 

### 2.5. Evaluation of mRNA Expression of Pro-Inflammatory Cytokines

DECs and HTR8/SVneo cells (3 × 10^5^ cells/well) were cultured in 12-well plates and then incubated with ZnO for 48 h at 37 °C in 5% *v/v* CO_2_ incubator. Successively, cells were stimulated ON with 100 ng/mL TNF-α. Supernatants were then collected, centrifuged and stored at −80 °C, whereas cells were lysed for total RNA extraction. Total protein measurement was carried out for each well by Bradford assay. Total RNA extracted was reverse transcripted using iScript cDNA Synthesis Kit (Bio-Rad, Milan, Italy). RT-qPCR was performed by Rotor-Gene 6000TM (Corbett, Explera, Ancona, Italy), using iQ TM SYBR Green Supermix (Bio-Rad, Milan, Italy). The melting curve was recorded between 55 °C and 99 °C, with a hold every 2 s. The relative amount of the gene of interest in each sample was normalized to the housekeeping gene 18 S and expressed as arbitrary units (AU), considering resting cells as a calibrator. The expression of several inflammatory cytokines was evaluated (TNF-α, IL-8, monocyte chemoattractant protein-1, MCP-1 and IL-6). Sequences and melting temperature of the primers used for RT-qPCR analysis are reported in Table 1.

### 2.6. Evaluation of IL-8 Secretion by ELISA

The amount of cytokine released in the supernatant of cultured DECs was measured by ELISA, with the Instant ELISA human IL-8 kit (Bender MedSystems, Milan, Italy) and with the RayBio^®^ Human IL-6 ELISA kit (RayBiotech, Peachtree Corners, GA, USA), following the manufacturer’s instructions. 

### 2.7. Statistical Analysis

Non-parametric tests (Mann-Whitney test) were carried out as data and were not normally distributed. Significance was set at *p* ≤ 0.05. Statistical analyses were performed using GraphPad Prism version 4.0 (GraphPad Inc., San Diego, CA, USA). 

## 3. Results

### 3.1. Definition of the Optimal Concentration of ZnO for In Vitro Analysis

In order to define the optimal concentration of ZnO to be used in culture, we evaluated the bioavailability, absorption and peak plasma of this molecule [24,25]. For this reason, we chose the concentration of 15 µg/mL. We ascertained also that ZnO addition at this concentration did not result in visible precipitates in the culture medium and was not responsible for pH variation of cell culture media. We defined that the treatment with 15 µg/mL ZnO did not significantly alter the medium pH and did not interfere with cell viability. In fact, cell viability experiments on DECs treated with different concentrations of ZnO (5, 15, 50 µg/mL) showed that only the highest concentration significantly affected cell viability (Figure S1). 

### 3.2. ZnO Exerted Anti-Inflammatory Effect Reducing the Expression of VCAM-1 by TNF-α Stimulated DECs

**Figure 2:**
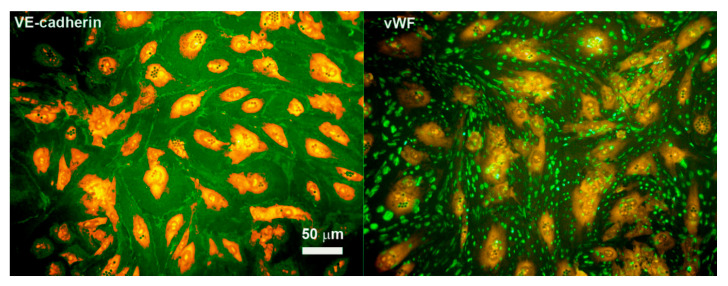
Characterization of decidual endothelial cells (DECs) by immunofluorescence. DECs isolated from first trimester human decidua were labelled with FastDiI (in red) and stained with VE-Cadherin or von Willebrand factor (vWF) (in green). The cells resulted 100% positive for the expression of these pan-endothelial cell markers. The images were acquired by microscope Leica DM3000. Original magnification 200×, scale bar, 50 μm.

Since ECs are involved in the orchestration of the inflammatory process at tissue level [26], we initially sought to inquire whether ZnO was able to exert an anti-inflammatory effect on these cells. We therefore used ECs isolated from normal human first trimester deciduas from healthy volunteers’ abortive tissue (DECs). These cells were characterized for the expression of classical endothelial markers by immunofluorescence. As shown in Figure 2, all cells resulted positively stained for VE-cadherin and vWF, confirming their endothelial origin. 

**Figure 3:**
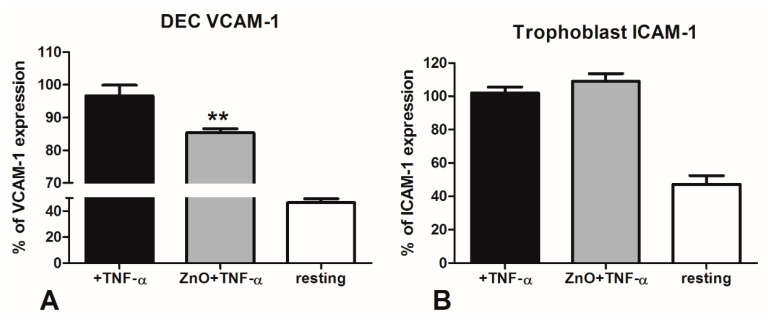
Analysis of vascular cell adhesion molecule-1 (VCAM-1) or intracellular adhesion molecule-1 (ICAM-1) expression on TNF-α stimulated placental cells. (**A**) Three different populations of DECs were incubated with 15 µg/mL zinc oxide (ZnO) for 48 h and successively stimulated overnight (ON) with TNF-α. The expression of VCAM-1 was evaluated by an enzyme-linked immunosorbent assay (ELISA) on whole cells. Data are expressed as mean ± SE. ** *p* < 0.05, as compared to the untreated (Mann-Whitney test). The “+TNF-α” condition represents the VCAM-1 expression of DECs after stimulation with TNF-α, whereas resting condition is representative of the cells without any treatment. We considered the untreated “+TNF-α” condition as our reference value, indicated as 100%. (**B**) HTR8/Svneo were incubated with 15 µg/mL ZnO, successively stimulated ON with TNF-α and incubated with anti-human ICAM-1. Data are expressed as mean ± SE results from five experiments, each performed in triplicate. The Mann-Whitney test indicated that there are no statistical differences (ns) between ZnO treated and untreated cells. The untreated “+TNF-α” condition represents the ICAM-1 expression of trophoblast cells after stimulation with TNF-α; we considered this condition as our reference value, indicated as 100%.

To evaluate the inflammatory response, DECs were grown to confluence and then stimulated with the pro-inflammatory cytokine TNF-α, to induce the expression of the adhesion molecule VCAM-1 on the cell surface [27]. VCAM-1 expression was evaluated by ELISA on whole cells. We analyzed the expression of VCAM-1 on resting DECs or DECs stimulated for 18 h with TNF-α. We compared this response with cells stimulated with TNF-α, previously pre-incubated for 48 h with ZnO. The results in Figure 3A clearly showed that ZnO was able to induce a significant downregulation of the VCAM-1 expression by DECs (about 35%). 

### 3.3. Analysis of the ICAM-1 Expression by First Trimester Trophoblast Cells Stimulated with TNF-α

We evaluated the anti-inflammatory effect of ZnO also on trophoblast cells, thereby repeating the previous experiments on the HTR8/Svneo cell line. The cells, after the incubation with ZnO, were stimulated ON with the pro-inflammatory cytokine TNF-α. HTR8/Svneo stimulated with TNF-α showed a strong increase in the expression of ICAM-1. In this case, we did not observe a significant inhibition of ICAM-1 expression by HTR8/Svneo, after incubation with ZnO (Figure 3B). 

### 3.4. ZnO Modulated the Pro-Inflammatory Response of DECs, Reducing Their Expression of Inflammatory Cytokines 

**Figure 4:**
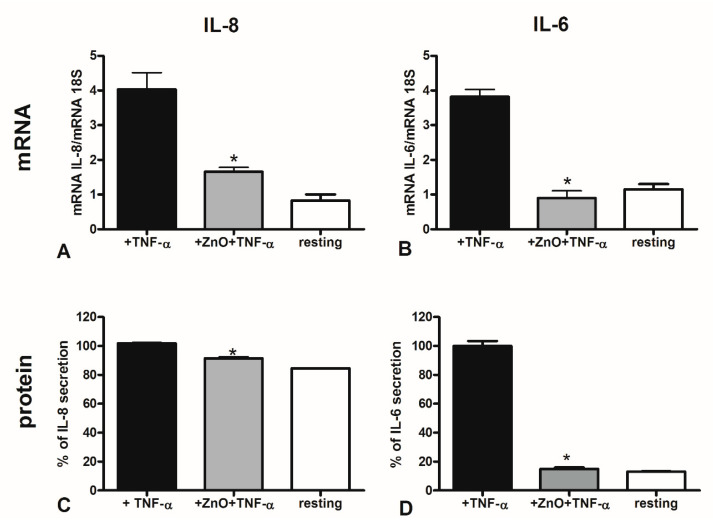
Expression of pro-inflammatory cytokines by DECs treated with zinc oxide (ZnO). RT-qPCR analysis for the expression of *IL-8* (**A**) and *IL-6* (**B**) highlighted a decreased mRNA expression of cytokines after treating the cells with ZnO and TNF-α, as compared to untreated cells (+TNF- α only). Data represent the mean ± S.E.M. of three different DEC populations. * *p* < 0.05 vs. Untreated (Mann-Whitney test). ELISA assays for the protein measurement of IL-8 (**C**) and IL-6 (**D**) in DECs’ supernatant highlighted a decreased protein expression level of cytokines after treating the cells with ZnO and TNF-α, as compared to untreated cells (+TNF- α only). Data represent the mean ± S.E.M. of three different DEC populations. * *p* < 0.05 vs. Untreated (Mann-Whitney test).

Since the excessive pro-inflammatory state occurring in adverse pregnancy outcome may be, to a certain extent, due to an enhanced production of pro-inflammatory cytokines, we tested the capability of the dietary supplement of ZnO to reduce the mRNA expression of IL-8, TNF-α, IL-6 and MCP-1. DECs were treated with ZnO for 48 h and then stimulated ON with TNF-α. Supernatants were collected for ELISA assay and cells were lysed for total mRNA extraction. We considered the resting condition as our reference value, indicated as AU. As visible in the graphs in Figure 4, ZnO was able to reduce the secretion of all the investigated cytokines by DECs, leading to resting levels, for IL-8 and IL-6, both in terms of mRNA (Figure 4A,B) and protein (Figure 4C,D) expression. The results are expressed as percentage of expression considering the untreated cells (cells stimulated with TNF-α, but not with ZnO), as 100% of cytokine secretion. At mRNA level, *MCP-1* and *TNF-α* expression also seemed to be downregulated after treatment with ZnO (Figure S2). We also confirmed the downregulation of the gene expression level of *IL-6* for HTR8/Svneo (Figure S3). 

## 4. Discussion

Despite being a physiological condition common to all pregnancies, inflammation at the placental level should be tightly regulated to prevent tissue injury potentially associated with pre-eclampsia (PE), preterm birth and foetal growth restriction (FGR). Women affected by pregnancy disorders exhibit an enhanced inflammatory state, as well as elevated levels of pro-inflammatory cytokines, such as TNF-α and IL-6, systemically and locally in the placenta [28]. Inflammation can be associated with deficient spiral artery remodeling and altered utero-placental perfusion dysfunction intrinsic to FGR/PE [29,30]. 

Being demonstrated that daily oral supplementation with zinc induce a significant rise in serum zinc levels [31] and that it exerts beneficial effects in the control of local and/or systemic inflammation, possessing immune-modulatory properties; ZnO appears as a potential molecule for the systemic treatment and prevention of inflammation during pregnancy [32,33]. 

Since no information is available about the effect of ZnO on human placental cells, we set up two different models by using both the HTR8/SVneo trophoblast cell line and DECs. First, we evaluated the effect of ZnO on the expression of adhesion molecules by HTR8/SVneo, characterized by the behavior of invasive EVT, and in particular, the adhesion molecule ICAM-1, a glycoprotein expressed by cytotrophoblast and readily inducible by cytokines stimulation [8]. Our results showed that ZnO was not able to reduce the expression of ICAM-1 by trophoblast cells stimulated with TNF-α. However, we observed a downregulation of the pro-inflammatory cytokine IL-6 expression. 

The most interesting data were obtained with DECs, since ZnO demonstrated the ability to exert anti-inflammatory effects in the reduction of VCAM-1 expression by TNF-α stimulated DECs. VCAM-1 is a 110-kD member of the immunoglobulin gene superfamily, representing an important inflammatory marker expressed on the surface of stimulated ECs. This cell surface protein serves as an inducible adhesion receptor for circulating mononuclear leukocytes [34]. 

The results obtained in the current study also demonstrated that ZnO was able to modulate the pro-inflammatory response of DECs by reducing their expression of inflammatory cytokines, such as IL-6, TNF-α, MCP-1 and IL-8. We appraise these results of great interest, considering that we have previously shown that DECs are programmed for controlling the inflammatory response at the feto-maternal interface [35]. DECs are characterized by low responsiveness to LPS stimulation in terms of IL-6, CXCL8 and CCL2 production, low expression levels of TLR4 and a strong constitutive activation of the non-canonical NF-κB pathway [6]. Taking into account these observations, the food supplementation of ZnO seems to contribute to endothelial cell function in the control of the inflammatory response. 

The differences in terms of adhesion molecules obtained with these two cell models reflect the distinctive functions played by these molecules at the placental level. The expression of adhesion molecules on the surface of endothelial cells serves as adhesion receptors for circulating leukocytes. The expression of the adhesion molecule on invading trophoblast are more related to the invasive process of the decidua. 

Another important aspect to consider is the characterization of specialized transporters on the surface of placental cells. Different isoforms of these transporters are necessary for the import and export of ZnO through the cell membrane [36] and no information are available about the distribution of these transporters in normal and pathological placentae. 

We therefore noted that, in both cases, ZnO induced a reduction of cytokine production. These results are in line with the observation about its immunomodulatory capacity previously made by other authors. In fact, it has been demonstrated that the reduced level of circulating Zn observed in elderly patients correlates with increased IL-6, IL-8 and TNF-α serum levels [37]. In addition, Zn deficiency seems to affect all aspects of the immune system, indicating that the availability of Zn is essential for the proper development and function of the immune system, even if some mechanisms of action are not yet decrypted; as a consequence, several pathologies appear to be characterized by imbalanced Zn homeostasis, such as cardiometabolic diseases [38], age-related diseases [39] and type II diabetes mellitus [40].

Emerging evidence from clinical and epidemiological studies greatly emphasizes the impact of ante-natal supplementation with multiple micronutrients in fetal growth without side effects [9]. Furthermore, there are already clinical data in literature supporting the importance of ZnO for pregnancy outcomes. It has been shown that growth retardation and cell-mediated immune dysfunction are major clinical effects in human of zinc deficiency [41], although several meta-analysis seemed to contradict this evidence. A very exhaustive meta-analysis conducted in 2015, including 21 randomized controlled trials reported in 54 papers, showed that insufficient evidence was available about other useful and important benefits exerted by zinc supplementation during pregnancy. Since it is also important to consider that the association with preterm birth may be a reflection of poor nutrition, an urgent priority will be to develop studies about the improvement of the overall nutritional status of disadvantaged populations, together with the interest in micronutrient and/or zinc supplementation [42]. 

## 5. Conclusions

Our data demonstrated that ZnO is able to reduce trophoblast and decidual endothelial response to inflammation. We can conclude that this dietary supplement might have a potential clinical value in the control of the inflammatory processes at the feto-maternal interface. Moreover, we can speculate that Zn homeostasis imbalance could be involved in pregnancy disorders. Further studies are now needed to clarify the role of ZnO in physiological and pathological pregnancies.

## Figures and Tables

**Table 1:** Primer used for RT-qPCR analysis.

Gene	Tm	Sense	Sequence	Accession Number
*18S*	60	Forward	ATCCCTGAAAAGTTCCAGCA	NM_022551.2
		Reverse	CCCTCTTGGTGAGGTCAATG	
*IL-8*	60	Forward	AGGTGCAGTAGTTTTGCCAAGGA	NM_000584.3
		Reverse	TTTCTGTGTTGGCGCAGTGT	
*TNF-α*	66	Forward	GGCCCAGGCAGTCAGATCAT	NM_000594.3
		Reverse	GGGGCTCTTGATGGCAGAGA	
*IL-6*	60	Forward	GTACATCCTCGACGGCATC	NM_000600.3
		Reverse	CCAGGCAAGTCTCCTCATTG	
*MCP-1*	60	Forward	ATCAATGCCCCAGTACC	NM_002982
		Reverse	AGTCTTCGGTAGTTTGGG	

Abbreviations: 18S ribosomal RNA (18S), interleukin (IL), tumor necrosis factor-α (TNF-α), monocyte chemoattractant protein-1 (MCP-1), melting Temperature (Tm).

## Supplementary Materials

The following are available online at https://www.mdpi.com/2072-6643/12/6/1822/s1. Figure S1: Cell viability evaluation after treating DECs with ZnO. DECs were treated with different concentration of ZnO (5-15-50 µg/mL). Cell morphology (A) was observed under Leica DMIL inverted microscope (Leica Microsystem) and images were collected using a Canon Powershot A640 digital camera (Canon). Cell viability (B) was evaluated by the addition of water soluble tetrazolium-1 (WST-1) reagent and the plate was read at 450 nm with a spectrophotometer, highlighting a significantly reduced cell viability with 50 µg/mL of ZnO. * *p* < 0.05 as compared to cells not treated with ZnO. Figure S2: Expression of pro-inflammatory cytokines by DECs treated with ZnO. RT-qPCR analysis for the expression of *MCP-1* (A) and *TNF-α* (B) highlighted a decreased mRNA expression of cytokines after treating the cells with ZnO and TNF-α, as compared to untreated cells (+TNF-α only). Data represent the mean ± S.E.M. of three different DEC populations. * *p* < 0.05 vs. Untreated (Mann-Whitney test). Figure S3: RT-qPCR analysis for the expression of IL-6 mRNA by HTR8/Svneo trophoblast cell line. Data represent the mean ± S.E.M. of three independent experiments run in duplicates. * *p* < 0.05 vs. Untreated. (Mann-Whitney test).
