# Supplementary figures and images for: Zinc Oxide Exerts Anti-Inflammatory Properties on Human Placental Cells
Source: Nutrients. 2020 Jun 18;12(6):1822. doi: 10.3390/nu12061822 (PMC7353449; doi:10.3390/nu12061822)

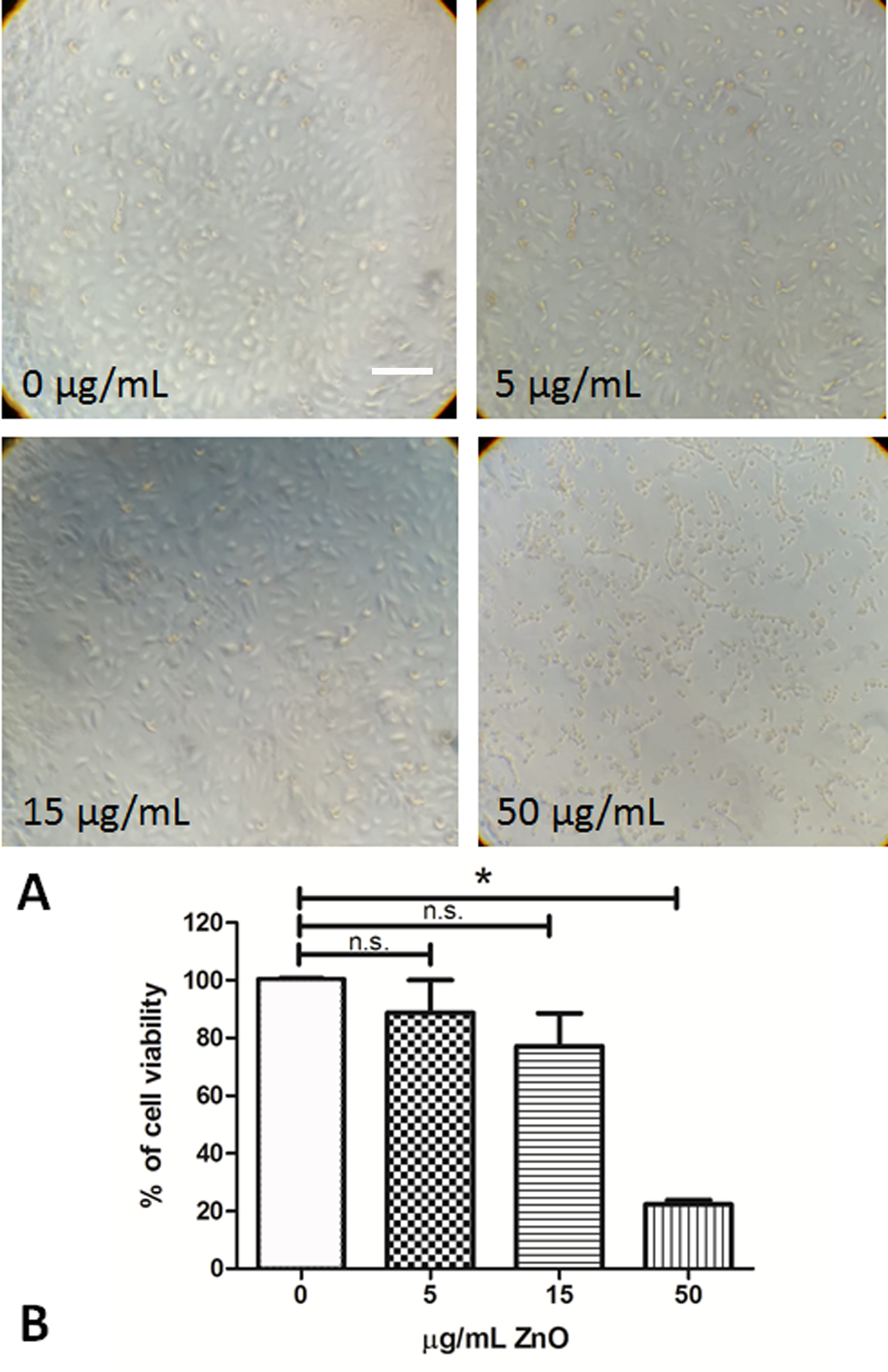

Supplement: Supplementary file 1 [file nutrients-12-01822-s001.zip › Supplemental Figures/Supplemental Figure 1.tif]

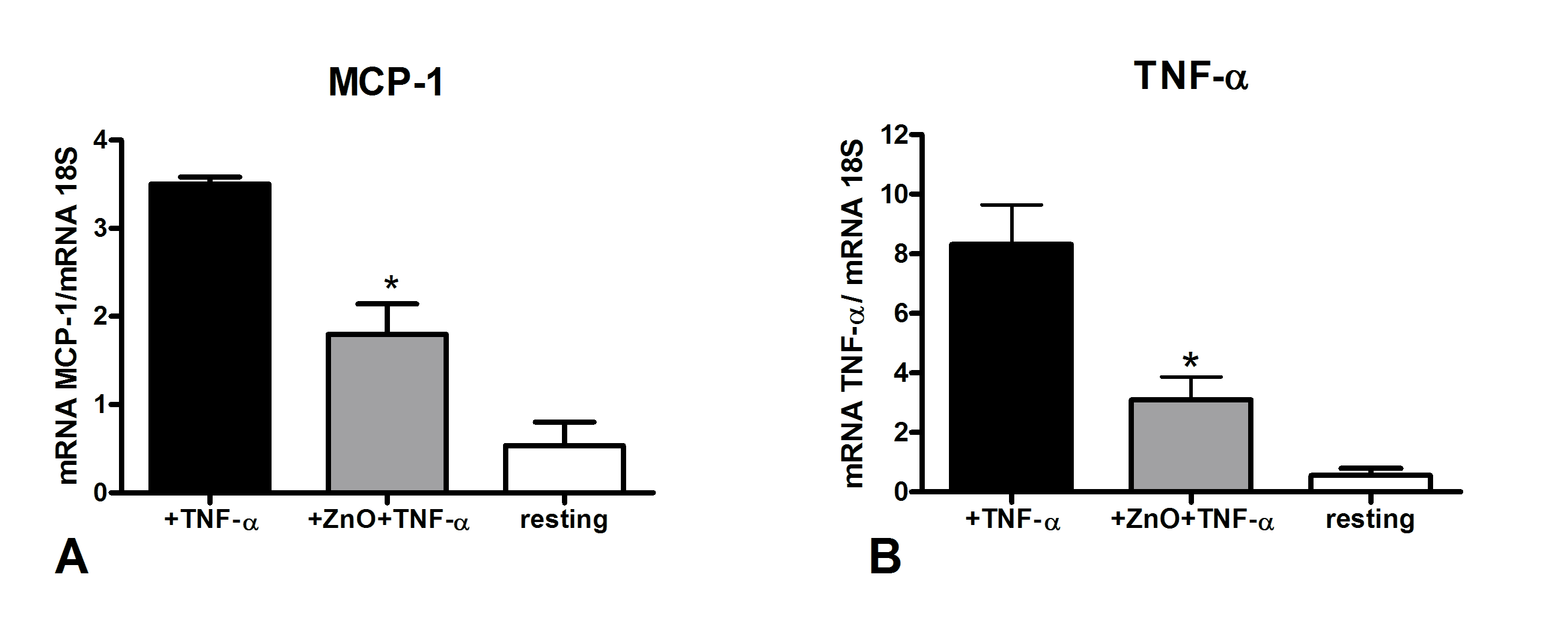

Supplement: Supplementary file 1 [file nutrients-12-01822-s001.zip › Supplemental Figures/Supplemental Figure 2.tif]

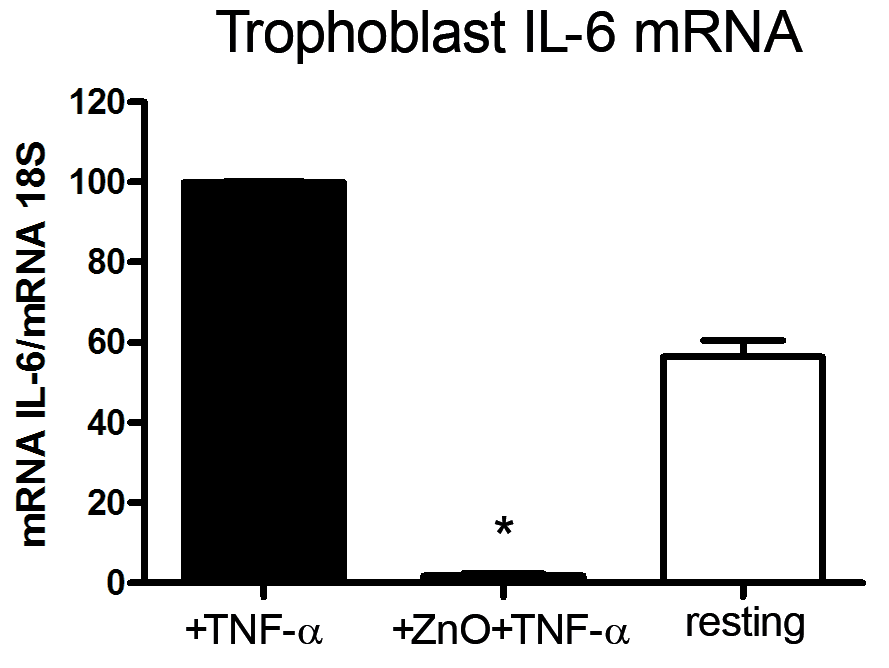

Supplement: Supplementary file 1 [file nutrients-12-01822-s001.zip › Supplemental Figures/Supplemental Figure 3.tif]
